# Conjugates of Chitosan with β-Cyclodextrins as Promising Carriers for the Delivery of Levofloxacin: Spectral and Microbiological Studies

**DOI:** 10.3390/life13020272

**Published:** 2023-01-18

**Authors:** Irina M. Le-Deygen, Anna A. Skuredina, Polina V. Mamaeva, Ilya M. Kolmogorov, Elena V. Kudryashova

**Affiliations:** Department of Chemical Enzymology, Lomonosov MSU, 119991 Moscow, Russia

**Keywords:** chitosan, cyclodextrin, drug delivery system, guest–host complexes, levofloxacin

## Abstract

In this work, we synthesized chitosan 5 kDa conjugates with β-cyclodextrins with various substituents as promising mucoadhesive carriers for the delivery of fluoroquinolones using the example of levofloxacin. The obtained conjugates were comprehensively characterized by spectral methods (UV-Vis, ATR-FTIR, 1H NMR, SEM). The physico-chemical properties of the complex formations were studied by IR, UV, and fluorescence spectroscopy. The dissociation constants of complexes with levofloxacin were determined. Complexation with conjugates provided four times slower drug release in comparison with plain CD and more than 20 times in comparison with the free drug. The antibacterial activity of the complexes was tested on model microorganisms Gram-negative bacteria *Escherichia coli* ATCC 25922 and Gram-positive *Bacillus subtilis* ATCC 6633. The complex with the conjugate demonstrated the same initial levofloxacin antibacterial activity but provided significant benefits, e.g., prolonged release.

## 1. Introduction

According to the World Health Organization, severe infections like pneumonia are one of the most challenging problems for international healthcare. Despite the significant progress in drug design and the development of drug delivery systems (DDS), there is a lack of effective treatment that ensures high antibacterial and anti-inflammatory activity and few side effects [1]. The inhaled method of administration is a promising technique to deliver drugs to the lungs with lower dosage and higher effectiveness in comparison with peroral and parenteral methods [2]. Fluoroquinolones, e.g., levofloxacin, are antibacterial drugs with a wide spectrum of action; these drugs are usually included in treatment protocols; however their bioavailability in the target tissues is low, and thus inhaled administration for this drug class is promising for the treatment of severe infections [3].

Development of biopolymer-based DDS is a promising approach, allowing fine tailoring of drug formulation properties. Usually, polymers for inhaled DDS must be mucoadhesive to provide higher affinity to the lung tissue [4,5]. One of the most promising polymers in these terms is chitosan, produced from chitin via diacylation [6,7]. The physical and chemical properties of chitosan strongly depend on the molecular weight and the diacylation degree [8], while unbounded amino groups are attractive targets for following chemical modification [9]. Chitosan with low molecular weight, e.g., 5 kDa, is considered as the most promising formulation for biomedicine, as it possesses the best solubility at neutral pH and provides for chemical modifications [10]. However, chitosan by itself has a weak affinity to antibiotic drugs like levofloxacin; thus, its application as a DDS for levofloxacin is still limited. Chitosan by itself serves as a prospective DDS for variable types of therapeutics, such as antiviral drugs and materials for tissue engineering [11,12,13], as well as in combination with other DDS like niosomes and liposomes [9,14].

On the other hand, one of the most promising carriers for fluoroquinolones is β-cyclodextrins (CD), torus-shaped oligosaccharides consisting of seven glucose molecules [15]. CDs form noncovalent guest–host complexes with fluoroquinolones [16] and are approved by the FDA for pharmaceutical applications. CD properties depend on the nature of substituents; however, CD complexes are not stable enough for inhaled administration and do not possess mucoadhesive properties [17,18].

Conjugates of chitosan and cyclodextrins combine the advantages of DDS [19] and could be considered as suitable drug delivery systems for antioxidants like idebenone [20], small peptides [21], and even proteins [22]. For severe lung infections, this type of conjugate seems very promising because it provides the mucoadhesive properties of the chitosan chain and the high drug loading efficacy of cyclodextrins. However, fine tailoring of this type of combined DDS is required for antibacterial drugs, as the properties of cyclodextrin, e.g., type of substituent, could significantly influence the complex formation efficacy and thus antibacterial activity [23].

In this work, we aimed to study two β-CD: with the amino group in the back chain (mono-(6-(hexamethylenediamine)-6-deoxy)-β-cyclodextrin) and 2-hydroxypropyl-β-cyclodextrin. The first one is considered as a cationic ligand, and the second is described in the literature as a carrier with a non-polar hydrophobic substituent [24].

Thus, in this work, we report on the development of the new drug carrier for inhaled administration on the bases of the conjugate chitosan–CD. We aimed to synthetize the conjugates and various substituents of CD to determine the most promising complex. Following the synthesis, we studied in detail the physico-chemistry of the levofloxacin binding with conjugates and determined the most effective complex. For the most effective complex, we aimed to study the antibacterial activity on the Gram-positive and Gram-negative model microorganisms *E. coli* and *B. subtilis*.

## 2. Materials and Methods

### 2.1. Materials

The following materials were used: chitosan oligosaccharide M_w_ 5000, >90% deacetylated, levofloxacin, trinitrobenzenesulfonic acid (TNBS), mono-(6-(hexamethylenediamine)-6-deoxy)-β-cyclodextrin (NH_2_-CD), 2-hydroxypropil-β-cyclodextrin (Hp-CD)–Sigma Aldrich (St. Louis, MO, USA); sodium phosphate buffer solution tablets Paneco (Moscow, Russia); *p*-toluenesulfonyl chloride (TsCl), Merck (Darmstadt, Germany); Acetic acid, acetonitrile, DMF, NaOH–Reakhim (Russia, Moscow).

*Escherichia coli* ATCC 25,922 and *Bacillus subtilis* ATCC 6633 are both from the Russian National Collection of Industrial Microorganisms (VKPM), Scientific Center, Kurchatov Institute (Moscow, Russia).

### 2.2. Synthesis of –O-p-toluenesulfonyl-mono-(6-(hexamethylenediamine)-6-deoxy)-β-cyclodextrin and O-p-toluenesulfonyl-2-hydroxypropyl-β-cyclodextrin (Ts-NH_2_-CD and Ts-Hp-CD)

*O-p*-toluenesulfonyl-mono-(6-(hexamethylenediamine)-6-deoxy)-β-cyclodextrin and *O-p*-toluenesulfonyl-2-hydroxypropyl-β-cyclodextrin (Ts-NH_2_-CD and Ts-Hp-CD) were synthesized according to an established procedure [25,26]. Twofold excess of a solution of TsCl in acetonitrile was added to an alkaline solution of β-CD derivatives (NH_2_-CD and Hp-CD, respectively), and a light brown precipitate was formed. Next, the reaction mixture was stirred at room temperature for 2 h, and the product was purified in Orange Scientific dialysis bags with a cut-off molecular weight of 2 kDa against distilled water for 12 h.

### 2.3. Synthesis of Conjugates: Chitosan–Cyclodextrin Derivatives

Solutions of Ts-NH_2_-CD and Ts-Hp-CD obtained in the first stage in DMF were added dropwise to a solution of chitosan in acetic acid pH 4.0 (molar concentration 3.45 mM). The reaction mixture was kept at 95 °C for 16 h, after which the products NH_2_-CD-Chit and Hp-CD-Chit were dialyzed in Orange Scientific dialysis bags with a cut-off molecular weight of 12–14 kDa against distilled water for 2 days.

### 2.4. Conjugate Characterization

The products NH_2_-CD-Chit and Hp-CD-Chit were characterized by titration of free amino groups with 2,4,6-trinitrobenzenesulfonic acid according to [27]. TNBS solution was added to solutions of conjugates and chitosan in a borate buffer solution (pH = 9.0); the optical density was recorded at a wavelength of 420 nm for 60 min on a Cary Eclipse UV-Vis spectrometer in a Hellma quartz cuvette Analytics at 22 °C. The initial concentration of TNBS during the titration of chitosan was 1 × 10^−3^ M; during the titration of conjugates 2 × 10^−3^ M, the concentration of polymers was 2 mg/L.

### 2.5. Levofloxacin Conjugate Complex Preparation

To the solution of levofloxacin in hydrochloric acid (pH 4.0, 3 mg/mL), the required amount of a solution of a β-cyclodextrin derivative with the same pH was added, and the volume was adjusted to 0.5 mL. The concentration of levofloxacin was maintained constant in all samples and was 1 mg/mL; the molar excess of the β-CD derivative ranged from 0.1 to 10. The complexes were incubated at 37 °C for 60 min.

### 2.6. ATR-FTIR Spectroscopy

The ATR-FTIR spectra were recorded with a Tensor 27 Fourier-transform IR spectrophotometer (Bruker, Ettlingen, Germany) supplied with an MCT detector and a thermostat (Huber, Offenburg, Germany). The detector was cooled with liquid nitrogen. Measurements were carried out in a BioATR-II thermostatically controlled cell of the attenuated total inner reflection (ATR), produced by Bruker, Germany, with a single reflection ZnSe crystal at 22 °C. The system was blown off with the constant rate flow of dry air by a Jun-Air device (Muenster, Germany). A sample aliquot of 30 μL was placed in the ATIR cell, and the spectrum was recorded thrice in the interval of 4000 to 950 cm^−1^ and at the resolution of 1 cm^−1^. Next, 70-fold scanning and averaging were performed. The background was recorded in the same way. All measurements were triplicated. The spectra were analyzed with the Opus 8.0 program.

### 2.7. UV-VIS Spectroscopy

The UV spectra were recorded with an Ultrospec 2100 pro (Amersham Biosciences, Amersharm, UK) within a wavelength range of 200–400 nm in a 1 mL quartz cell (Hellma Analytics, Muellheim, Germany). The initial samples were diluted with HCl (pH = 4.0) to a levofloxacin concentration of 3 × 10^−5^ M. All measurements were triplicated.

### 2.8. Fluorescence Spectroscopy

The emission of fluorescence spectra of the complexes was recorded with a Varian Cary fluorescence spectrometer in the range from 350 to 650 nm at an excitation wavelength of λ_ex_ = 288 nm in a 1 mL quartz cell (Hellma Analytics). The initial samples were diluted with HCl (pH = 4.0) to a levofloxacin concentration of 3 × 10^−5^ M. All measurements were triplicated.

### 2.9. Scanning Electron Microscopy

The sample morphology was investigated using a JEOL JIB 4501 multibeam system (Freising, Germany). The acceleration voltage was set to 5 kV. The SEM micrographs were acquired in SE mode. Powder samples placed onto SEM slabs were coated by a 15 nm gold layer with the help of a Q150R Plus sputter coater from Quorum Technologies (Laughton, UK).

### 2.10. 1H NMR

A total of 20–25 mg of the sample was dissolved in D_2_O (~600 μL). 1H NMR spectra were recorded using a Bruker Avance 400 spectrometer (Ettlingen, Germany) with operating frequencies of 400 MHz. Chemical shifts (δ) in ppm were reported as quoted relative to the residual signals of D_2_O (4.79 ppm).

### 2.11. Kdis Calculation

The dissociation constant of the complexes according to the data obtained was calculated using the Benesi-Hildebrand equation [28], linearized in the coordinates 1/(A − A_0_) from 1/[L], where A_0_ is the intensity of the analytical signal of free levofloxacin, A is the intensity of the analytical signal of levofloxacin in the presence of ligand, [L] is the ligand concentration, and Kdis is the dissociation constant of the complex.

$$\frac{1}{\left(A-A_{0}\right)}=\frac{\mathrm{Kdis}}{\left(A_{\infty }-A_{0}\right)\times \left[L\right]}+\frac{1}{\left(A_{\infty }-A_{0}\right)}$$

### 2.12. Levofloxacin Release Kinetics Study

Studies were carried out in 0.1 mM HCl solution (pH 4.0). The solutions of NH_2_-CD–levofloxacin and Chit-NH_2_-CD–levofloxacin complexes were transferred into a dialysis capsule (Serva, Mw cut-off 2–4 kDa) and placed on a shaker at 37 °C at 120 rpm. Levofloxacin concentration in samples was 3 × 10^−5^ M, CD tori concentration in all samples was 1.5 × 10^−5^ M. During a period of 24 h, probes of external solution were analyzed with UV-Vis to evaluate levofloxacin concentration.

### 2.13. Antibacterial Activity Tests

*Escherichia coli* ATCC 25,922 and *Bacillus subtilis* ATCC 6633 were cultured in Luria Bertuni liquid growth medium for 12 h. These cultures were used for minimum inhibition concentration (MIC) determination. The agar well diffusion method was performed as a standard approach for the determination of the drug form’s MIC in vitro [29,30]. Briefly, four wells were cut in the agar by a sterile plastic pipette tip after the distribution of overnight culture over the agar surface. Welss 10 mm in diameter were filled with 100 μL of the sample: sterile buffer (negative control), levofloxacin, and levofloxacin–conjugate (levofloxacin concentration 0.1, 0.2, 0.5, and 1 μg/mL for both strains). C_CD’s torus in conjugate_ = C_Levofloxacin_. Then, the plates were placed in the incubator for 24 h at 37 °C. The inhibition zones appeared around the wells and their diameters were analyzed. MIC is assumed as the concentration at which the inhibition zone equals the area of the removed agar disk (d~11 mm) and is reported with standard deviation. Each sample was tested in triplicate.

### 2.14. Statistical Analysis

The reported data are presented as the mean value ± standard deviation (S.D.). The significance of differences was determined at the probability level of 0.05.

## 3. Results and Discussion

### 3.1. Conjugates: Chitosan–Cyclodextrin Synthesis

**Figure 1:**
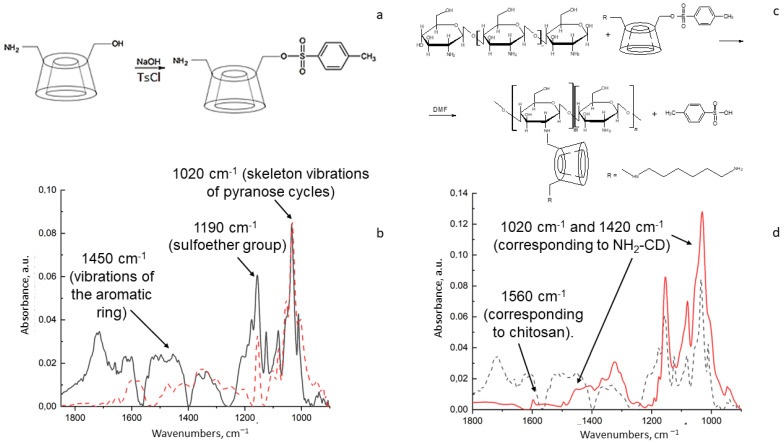
Synthesis of chitosan–β-cyclodextrin conjugates on sample NH_2_-CD-Chit. (**a**) Synthesis of Ts-NH_2_-CD. (**b**) ATR-FTIR spectrum of Ts-NH_2_-CD (solid black line) and NH_2_-CD (red dotted line) in water solution at 22 °C. (**c**) Synthesis of NH_2_-CD-Chit. (**d**) ATR-FTIR spectrum of NH_2_-CD-Chit (red solid line) and Ts-NH_2_-CD (dotted black line) in water solution at 22 °C.

We synthesized conjugates of chitosan with β-cyclodextrins by means of two-stage synthesis. At the first stage (Figure 1a), Ts-NH_2_-CD and Ts-Hp-CD were synthesized according to [25,26]. The main advantages of this technique are mild conditions and high yields: we achieved 37% and 42% yield, correspondingly. Products were light-brown amorphous precipitates that were slightly soluble in water.

On the ATR-FTIR spectrum of the compound obtained in the first stage (Figure 1b), absorption peaks are observed at the following wavenumbers: 1450 cm^−1^ (vibrations of the aromatic ring), 1190 cm^−1^ (sulfoether group) and 1020 cm^−1^ (skeleton vibrations of pyranose cycles) [31]. This spectral pattern corresponds to the expected one [32]. Compared to unmodified β-CD, the peak structure becomes more complex due to the appearance of peaks corresponding to the aromatic ring and the sulfoether group.

At the second stage (Figure 1c), NH_2_-CD-Chit and Hp-CD-Chit were synthesized via the S_N_2 nucleophilic substitution reaction. The amino group in chitosan is a stronger nucleophile than the hydroxyl groups [33]; therefore, it is this group that primarily attacks the partial positive carbon charge on β-CD. Since –OTs is a good leaving group, it is quickly replaced by the chitosan amino group in this reaction. As a result, NH_2_-CD-Chit and Hp-CD-Chit were obtained with a yield of 19% and 24%, correspondingly, with an amorphous, soluble-in-water substance that was light yellow in color.

In the ATR-FTIR spectrum (Figure 1d) of NH_2_-CD-Chit, the following bands were observed: 1020 cm^−1^, 1420 cm^−1^ (corresponding to mono-(6-(hexamethylenediamine)-6-deoxy)-β-cyclodextrin), and 1560 cm^−1^ (corresponding to chitosan). Chitosan peaks are very low-intensity and make an insignificant contribution against the background of intense CD peaks. Indicative of the effectiveness of the reaction is the absence of broad peaks in the range of 1800–1550 cm^−1^. This indicates that the product of the second stage was purified from the -OTs group and the reaction was completed [26]. According to previously published data, when the tosyl group is removed, the intensity in the region of 1800–1650 cm^−1^ decreases significantly. Moreover, the peak at 1200 cm^−1^ is smoothed out, which is in good accordance with –OTs group removal.

To determine the ratio of chitosan and cyclodextrin torus in conjugates, the obtained spectra (Figure 1d) were compared with the calculated spectra, and the analysis showed that the ratio of Chit:β-CD in the product was 1:10.

**Figure 2:**
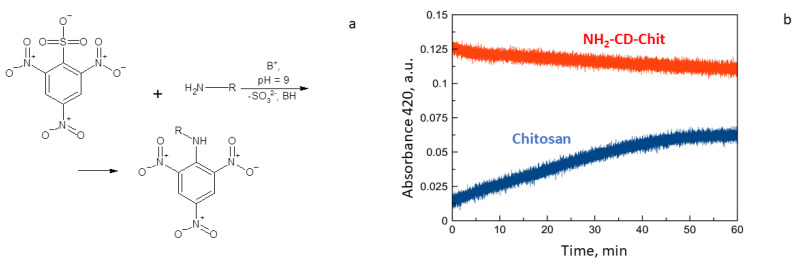
(**a**) Free amino group titration with TNBS. (**b**) Kinetic curves (420 nm) for chitosan solution (blue) and conjugate (red) NH_2_-CD-Chit. Borate buffer solution. pH 9.0.

To confirm the obtained data, we titrated free amino groups in conjugates with TNBS (Figure 2a). As a result of this reaction, the concentration of the colored product increased with time (Figure 2b). When titrating the conjugate, such a change in the concentration of the product was not observed; it was mostly independent of time. Hence, in the synthesized conjugates, there were no amino groups of chitosan available for binding; they were shielded by a cyclodextrin derivative.

1H NMR is a powerful method to study the structure of organic compounds; however, for polymers, e.g., in chitosan it is challenging to identify signals, and some key approaches have been published [34]. In the 1H NMR spectrum of Chit (Figure S1a), signals in the region 4.0–3.5 ppm were assigned to H2-H6, and 2.02 ppm correspond to NAc [35,36]. For NH_2_-CD-Chit, we observed the appearance of new signals that correspond to NH_2_-CD [37] (Figure S1b): 4.98 ppm was assigned to H1, and the peaks in the region 4.0–3.2 ppm corresponded to H2–H5 of the D-glucopyranose units; the most intense bands in the region 3.0–2.5 ppm were assigned to methylene protons. Thus, NMR confirms the NH_2_-CD-Chit conjugate synthesis.

**Figure 3:**
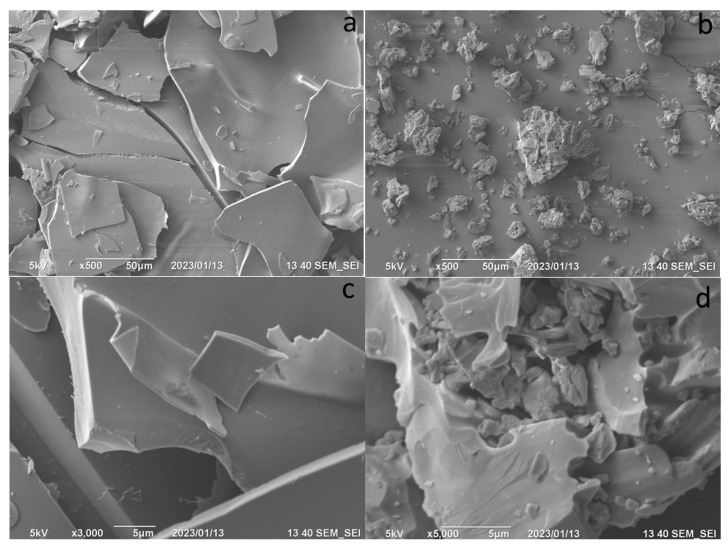
Scanning electron microscopy for NH_2_-CD (**a**,**c**) and NH_2_-CD-Chit (**b**,**d**). The acceleration voltage was set to 5 kV.

To determine the differences between the two types of levofloxacin carrier, NH_2_-CD-Chit conjugate and initial NH_2_-CD, we compared the morphology of samples (Figure 3). For NH_2_-CD, smooth surfaces were observed (Figure 3a,c), so the sorption of the drug was possible only in cyclodextrins tori. The conjugates (Figure 3b,d) were characterized by a much more developed surface, suitable for additional sorption of levofloxacin.

Thus, we synthetized conjugate of chitosan M_w_ 5 kDa with β-CD derivatives. On average, one molecule of the conjugate in the main chain has 28 units and carries 10 cyclodextrin torus (corresponding to the theoretical M_w_ 17.5 kDa for NH_2_-CD-Chit and 17 kDa for HP-CD-Chit), and the type of substituent does not influence the degree of modification. With the inclusion of levofloxacin in all tori of cyclodextrins, the calculated drug: carrier mass ratio is approximately 20%, which is suitable for further use as an inhalation delivery system [38].

$$\mathrm{Drug}:\mathrm{Carrier}\;\mathrm{ratio}=\frac{M_{\mathrm{levofloxacin}}\times 10}{M_{\mathrm{conjugate}}}\times 100\%$$

The conjugates have a higher solubility in comparison with chitosan and do not tend to form gels in neutral media and thus are suitable for the further studies.

### 3.2. Complex Formation of Levofloxacin with β-Cyclodextrin Derivatives

CDs serve as a host in noncovalent inclusion complexes with fluoroquinolones, and here we aimed to load levofloxacin into the CD torus in conjugates.

To create an efficient levofloxacin delivery system, we studied the physico-chemical properties of the guest–host complexes of the drug with CD derivatives. The size of the β-CD inner cavity corresponds to the size of levofloxacin’s rings [23,39] and allows us to study the effect of the nature of the substituent in β-CD on the dissociation constants of their inclusion complexes with levofloxacin.

**Figure 4:**
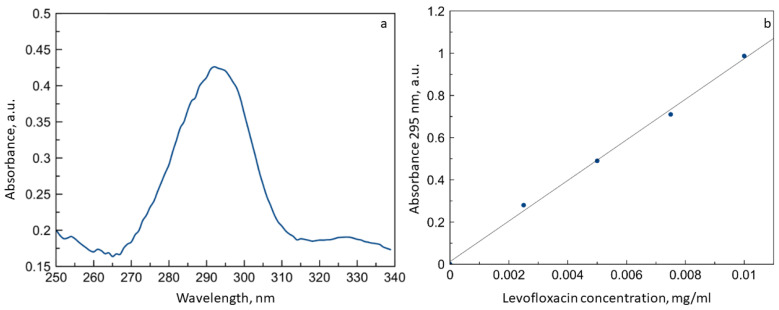
(**a**) UV spectrum of levofloxacin 3 × 10^−5^ M. (**b**) Dependence of absorbance 295 nm on the levofloxacin concentration. HCl solution, pH 4.0, T = 22 °C.

The UV spectrum of levofloxacin (Figure 4a) presents a pronounced absorption band with an intensity maximum λ_max_ = 295 nm, which corresponds to the absorption of the aromatic backbone of fluoroquinolone. This peak is relevant for the analysis of the content of the drug in solution (Figure 4b), and the limit of detection was determined as 0.001 mg/mL.

**Figure 5:**
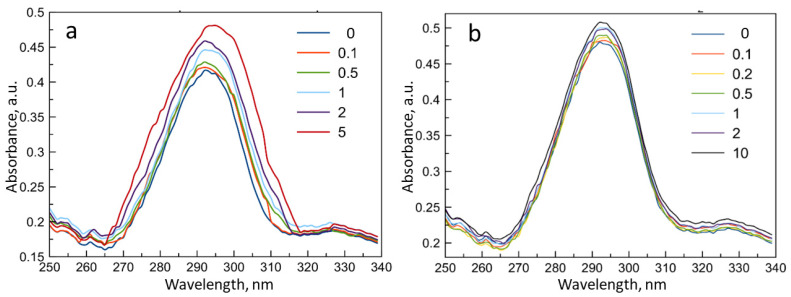
UV spectra reflecting the effects of complex formation of levofloxacin with β-cyclodextrin-containing ligands ((**a**)–Hp-CD and (**b**)–NH_2_-CD-Chit) in the molar ratio of levofloxacin: ligand 10:1, 5:1, 2:1, 1: 1, 1:2, 1:5, 1:10. The concentration of levofloxacin in all samples was 3 × 10^−5^ M, HCl solution, pH 4.0, T = 22 °C.

According to the previously published data [15], the binding of fluoroquinolones by β-CD derivatives is accompanied by a change in the molar absorption coefficient ε of fluoroquinolones. Here, we found that the complex formation between β-CD derivatives and levofloxacin leads to an increase of ε depending on the molar excess of cyclodextrin torus (Figure 5).

The dissociation constants of the complexes calculated using the Benesi-Hildebrand equation are presented in Table 1. The obtained values agree well, in the order of magnitude with the literature data for fluoroquinolone systems with other β-CD derivatives [24,40]. In addition, the dissociation constants with polymeric ligands are one order of magnitude lower than those for the monomeric ligands, indicating additional stabilization of the complex by polymer chains.

The complex formation of levofloxacin with the ligands NH_2_-CD-Chit and Hp-CD-Chit as a control was also investigated using fluorescent analysis, since it is characterized by high sensitivity and speed of analysis.

**Figure 6:**
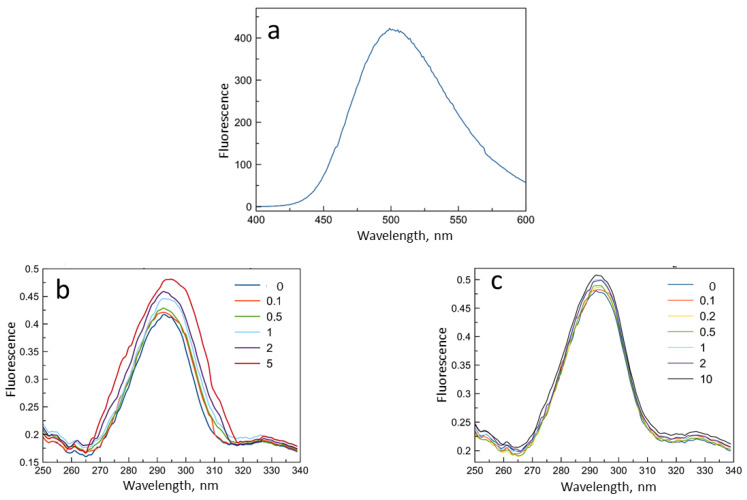
Spectra of fluorescence emission of levofloxacin. (**a**) Levofloxacin solution 3 × 10^−5^ M, pH = 4.0, T = 22 °C. (**b**,**c**) UV spectra reflecting the effects of complex formation of levofloxacin with β-cyclodextrin-containing ligands ((**b**) NH_2_-CD, (**c**) NH_2_-CD-Chit) in the molar ratio of levofloxacin: ligand 10:1, 5:1, 2:1, 1: 1, 1:2, 1:5, 1:10. The concentration of levofloxacin in all samples 3 × 10^−5^ M, pH = 4.0, T = 22 °C, λ excitation 288 nm.

The levofloxacin fluorescence spectrum (Figure 6a) displays an emission peak of 500 nm (λ excitation 288 nm), and the intensity of this band is also sensitive to the changes in the drug microenvironment [41]. With an increase in the concentration of all ligands, an increase in the intensity of the levofloxacin fluorescence peak was observed (Figure 6b,c). Linearization of the obtained data in the Benesi-Hildebrand coordinates provided data on the dissociation constants of levofloxacin complexes with β-CD-containing ligands. The values of the constants are presented in Table 1.

The data obtained from the fluorescence data are consistent with the results from the UV spectroscopy: the lowest values of dissociation constants corresponding to the most stable complex were achieved for complexes with polymeric ligands, and the ligand NH_2_-CD-Chit shows the lowest value of the dissociation constant. For a deeper study of the processes of complex formation, the ATR-FTIR spectroscopy was used, which provides a wide range of information about the microenvironment of the main functional groups of molecules in noncovalent complexes.

**Figure 7:**
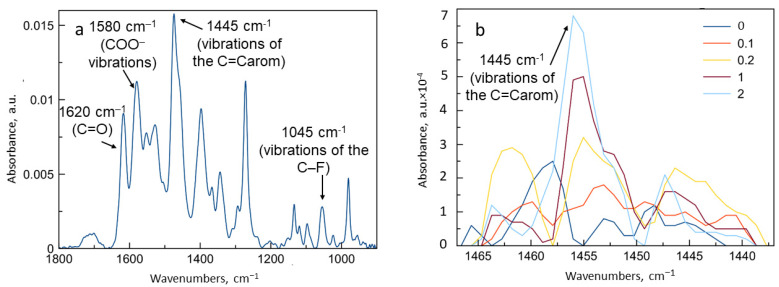
ATR-FTIR spectra of levofloxacin (**a**) and its complexes with NH_2_-CD-Chit (**b**) in variable molar ratio. Levofloxacin concentration 3 × 10^−5^ M, HCl solution, pH = 4.0, T = 22 °C.

On the ATR-FTIR spectrum of levofloxacin (Figure 7a), the most intensive absorption bands are 1445 cm^−1^, corresponding to vibrations of the C=Carom bond in the quinolone structure, and 1045 cm^−1^, due to vibrations of the C–F bond. The 1620 cm^−1^ band corresponds to the C=O stretching vibrations in the carbonyl group, and the 1580 cm^−1^ band corresponds to the deprotonated COO– [3].

ATR-FTIR spectroscopy was used to study the complex formation of levofloxacin with NH_2_-CD-Chit as the one with best dissociation constant. With an increase in the ligand excess, the intensity of the absorption band at 1455 cm^−1^ increased. Linearization of the sorption isotherm in the Benesi-Hildebrand coordinates leads to Kdis value (2.1 ± 0.5) × 10^−5^ M, which is in good agreement with the data obtained based on UV spectroscopy and fluorescence.

Thus, the complex formation of levofloxacin with conjugates based on β-cyclodextrin and chitosan derivatives is characterized by the values of dissociation constants (Table 1), which are of particular interest for the development of a drug delivery system.

The obtained values are in good agreement with the literature data (same order with moxifloxacin and CD derivative). In the case of oligomeric ligands, the literature describes a decrease in the dissociation constant by one order of magnitude [41], and upon binding to a polymeric ligand, a similar decrease in the value of the dissociation constant also occurs due to the stabilization of the inclusion complex by polymer chains.

### 3.3. Levofloxacin Release Studies

It is well-known that formation of guest–host complexes with fluoroquinolones and cyclodextrins and its derivatives could significantly influence the rate of drug release.

**Figure 8:**
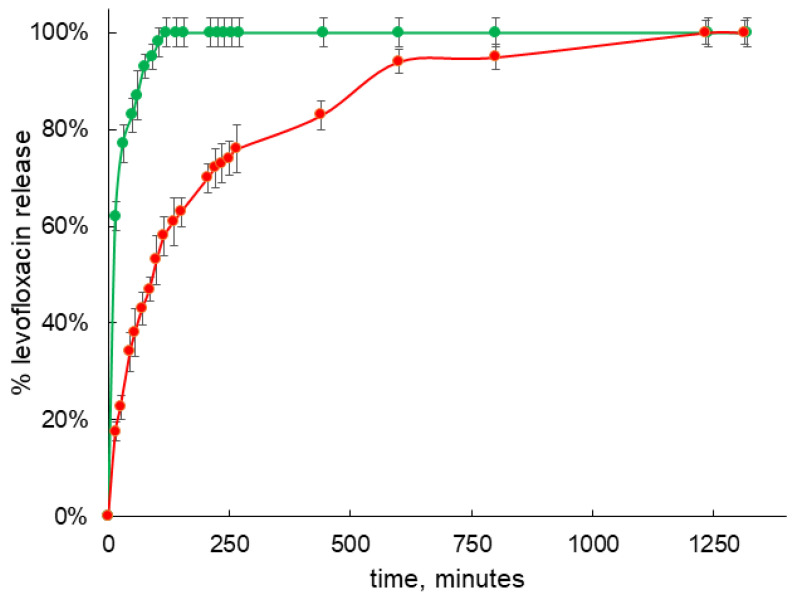
Levofloxacin release studies from complex with NH_2_-CD (green dots) and Chit-NH_2_-CD (red dots). Studies were carried out in 0.1 mM HCl solution (pH 4.0) at 37 °C 120 at rpm. Levofloxacin concentration in samples was 3 × 10^−5^ M, CD tori concentration in all samples was 1.5 × 10^−5^ M.

Since the conjugate based on NH_2_-CD has the highest binding efficiency for levofloxacin, it is the most promising as the carrier with prolonged release, so the release kinetic of levofloxacin was investigated by equilibrium dialysis and UV-VIS spectroscopy according to the technique previously described [42]. In separate experiment, we observed that free levofloxacin releases from the dialysis capsule in less than 35 min, which is in a good agreement with previously published data for other fluoroquinolones like moxifloxacin [42]. Formation of the guest–host complex with NH_2_-CD leads to slowing down the rate of release, so around 180 min is required to achieve almost 100% of drug release (Figure 8 green plot). On the other hand, the release from the guest–host of conjugate Chit-NH_2_-CD is significantly prolonged (Figure 8 red plot) and almost 100% of levofloxacin release is observed only after 800 h of incubation, so the rate is decreased more than four times in comparison with the complex with plain CD and more than 20 times in comparison with the free drug.

Why does the conjugate allow for a prolonged release of levofloxacin? The difference from the native host–guest complexes is caused by the influence of the oligomeric chain of chitosan. Loops of chitosan in an acidic environment are highly mobile [10], and levofloxacin literally “gets entangled” in the carrier matrix. This possibility is indirectly evidenced by the SEM data (Figure 3): the developed surface of the conjugate particles makes it possible to assume that in aqueous solutions the polymer will provide the matrix effect. We previously observed such a prolonged release of moxifloxacin upon the addition of a matrix for oligomeric particles of cyclodextrins [42]; Yuan et al. had similar results for the example of ketoprofen loaded to the high molecular weight chitosan–CD conjugates [25].

These significant changes provide us an opportunity to tailor properties of drug delivery systems and create a therapeutic form with prolonged release.

### 3.4. Antibacterial Activity Tests

**Figure 9:**
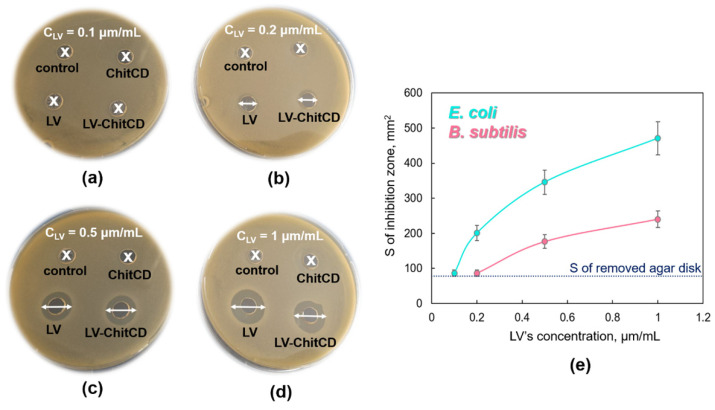
Inhibition zones appeared on Petri dishes, CLV = 0.1 μg/mL (**a**), 0.2 μg/mL (**b**), 0.5 μg/mL (**c**), and 1 μg/mL (**d**); C_conjugate_ = 0.5 μg/mL (**a**), 1 μg/mL (**b**), 2.4 μg/mL (**c**), 5 μg/mL (**d**), C_CD’s torus in conjugate_ = C_Levofloxacin,_ agar well diffusion method, B. subtilis ATCC 6633 pH 7.4 (0.02 M sodium phosphate buffer solution), 37 °C, 24 h of incubation. (**e**) The area of inhibition zones depending on CLV, *B. subtilis* ATCC 6633 and *E. coli* ATCC 25,922, agar well diffusion method, pH 7.4 (0.02 M sodium phosphate buffer solution), 37 °C, 24 h of incubation.

We studied the antibacterial activity of levofloxacin, NH_2_-CD-Chit, and levofloxacin–NH_2_-CD-Chit complexes against Gram-negative bacteria *Escherichia coli* ATCC 25,922 and Gram-positive *Bacillus subtilis* ATCC 6633 (Figure 9). Levofloxacin demonstrated concentration-dependent antibacterial action in vitro on both bacterial strains. Levofloxacin’s MIC *E. coli* and MIC *B. subtilis* are 0.1 μg/mL and 0.2 μg/mL, respectively, which is in good agreement with our recent data [23].

CDs is proven to be nontoxic in vitro [30], while unmodified chitosan, according to the literature data, possesses antibacterial activity against fungi, Gram-positive bacteria, and Gram-negative bacteria [43,44]. The modification of amino groups by CDs can change chitosan’s antibacterial properties: with the increase of immobilized CDs content, the polymer’s antibacterial properties decrease [45]. Thus, in our case, CDs decreased chitosan’s in vitro action pronouncedly.

The formation of chitosan–drug complexes might also change chitosan’s biological properties. For instance, clove essential oil loaded in chitosan nanoparticles demonstrated lower activity against *S. aureus*, *L. monocytogenes,* and *S. typhi* compared to the action of chitosan. This effect might be associated with the neutralization of polysaccharides’ positive charge via interaction with essential oil. Nevertheless, MIC values for oil and loaded particles were close, so chitosan did not decrease the oil’s in vitro properties [46].

For all studied concentrations, the levofloxacin–NH_2_-CD-Chit complex demonstrated antibacterial activity comparable to levofloxacin’s action. MIC (levofloxacin) = MIC (levofloxacin–NH_2_-CD-Chit) for both *E. coli* and *B. subtilis*. Thus, CDs immobilization on chitosan does not lead to the decrease of levofloxacin’s antibacterial activity on Gram-positive and Gram-negative bacteria.

## 4. Conclusions

In this work, we created a new delivery system for levofloxacin based on conjugates of low molecular weight chitosan and derivatives of β-cyclodextrins with various substituents: an amino group and a hydroxypropyl residue. Such systems may be promising for creating antibiotic therapy for inhaled administration. We synthesized, purified, and characterized conjugates of chitosan with β-cyclodextrins (HP-CD and NH_2_-CD) under mild conditions: there were about 10 tori per chitosan chain. At the same concentration, the conjugates dissolve in water faster and do not tend to form gels in neutral media, in contrast to unmodified olygochitosan. According to scanning electron microscopy, the conjugate particles have a developed surface, which makes them preferable for inhaled administration of levofloxacin.

The interaction of conjugates with levofloxacin due to the formation of host–guest complexes was studied by ATR-FTIR, UV-VIS, and fluorescence spectroscopy. Linearization of sorption isotherms in Benesi-Hildebrand coordinates made it possible to determine the dissociation constants of the complexes; the best order (10^−5^) was achieved for the levofloxacin- NH_2_-CD-Chit system.

Complexation of levofloxacin with the conjugate NH_2_-CD-Chit causes the most pronounced effect of the sustained release. It was found that the release of levofloxacin from the complex with the conjugate is approximately four times slower relative to the complex with cyclodextrin and approximately 20 times relative to free levofloxacin. Thus, the effect of the matrix can significantly affect the release.

The antibacterial activity of the systems was tested on Gram-positive and Gram-negative model microorganisms; it was found that binding into the complex does not lead to a loss of antibacterial activity. Indeed, prolonged release of levofloxacin may interfere with the results of antibacterial activity tests. The conjugate slows the drug’s release, which might lead to prolonged antibacterial action, and 24 h of in vitro experiment might not demonstrate all the potential of the conjugate.

We previously reported on the carrier for levofloxacin—a synthetic polymer based on cyclodextrins [23]. This type of carrier is able to enhance the antibacterial effect of levofloxacin, probably by extracting lipids or proteins from the bacterial membrane. For the system described in this paper, we have to unravel the nature of the interaction with bacterial cells and investigate its mucoadhesive properties.

As an alternative delivery system for inhaled levofloxacin, liposomal systems are considered, for example, in [47,48,49]. On the one hand, liposomes have a high biocapacity and allow a prolonged release of the contents; for example, in [47], the release of half of the loaded drug is observed after only 15 h. On the other hand, the production of liposomes and the control of their physico-chemical properties is a much more difficult task compared to the production of chitosan conjugates with cyclodextrin. Moreover, the drug: carrier mass ratios for liposomes loaded with levofloxacin range from 2 to 7% [47,49], while for complexes with conjugates herein, the calculated ratio reaches 20%. Thus, chitosan conjugates with cyclodextrin represent an undoubted interest for further research as a delivery system for antibacterial drugs.

The results obtained open up new prospects for the development of inhalation delivery systems for antibacterial drugs for the treatment of severe pneumonia.

## Figures and Tables

**Table 1:** Values of dissociation constants for complexes of levofloxacin with cyclodextrin-based carriers, as revealed by spectroscopic methods, M. The concentration of levofloxacin in all samples 3 × 10^−5^ M, pH = 4.0, T = 22 °C.

Method/Ligand	NH_2_-CD-Chit	Hp-CD-Chit	Hp-CD
UV spectroscopy	(4.8 ± 1.2) × 10^−5^	(4.8 ± 1.8) × 10^−5^	(2.2 ± 0.9) × 10^−4^
Fluorescence spectroscopy	(1.25 ± 0.80) × 10^−5^	(6.2 ± 1.1) × 10^−5^	(1.9 ± 0.3) × 10^−4^
ATR-FTIR spectroscopy	(2.1 ± 0.5) × 10^−5^	-	-

## Data Availability

The data presented in this study are available on request from the corresponding author. The data are not publicly available due to privacy.

## Supplementary Materials

The following supporting information can be downloaded at: https://www.mdpi.com/article/10.3390/life13020272/s1, Figure S1: 1H NMR spectra of Chit (a) and NH2-CD-Chit (b), D2O, 400 MHz.
