# Supplementary material for: Conjugates of Chitosan with β-Cyclodextrins as Promising Carriers for the Delivery of Levofloxacin: Spectral and Microbiological Studies
Source: Life (Basel). 2023 Jan 18;13(2):272. doi: 10.3390/life13020272 (PMC9960298; doi:10.3390/life13020272)
Supplement: Supplementary file 1 [file life-13-00272-s001.zip › life-2167040-supplementary.pdf]

Supplementary Materials

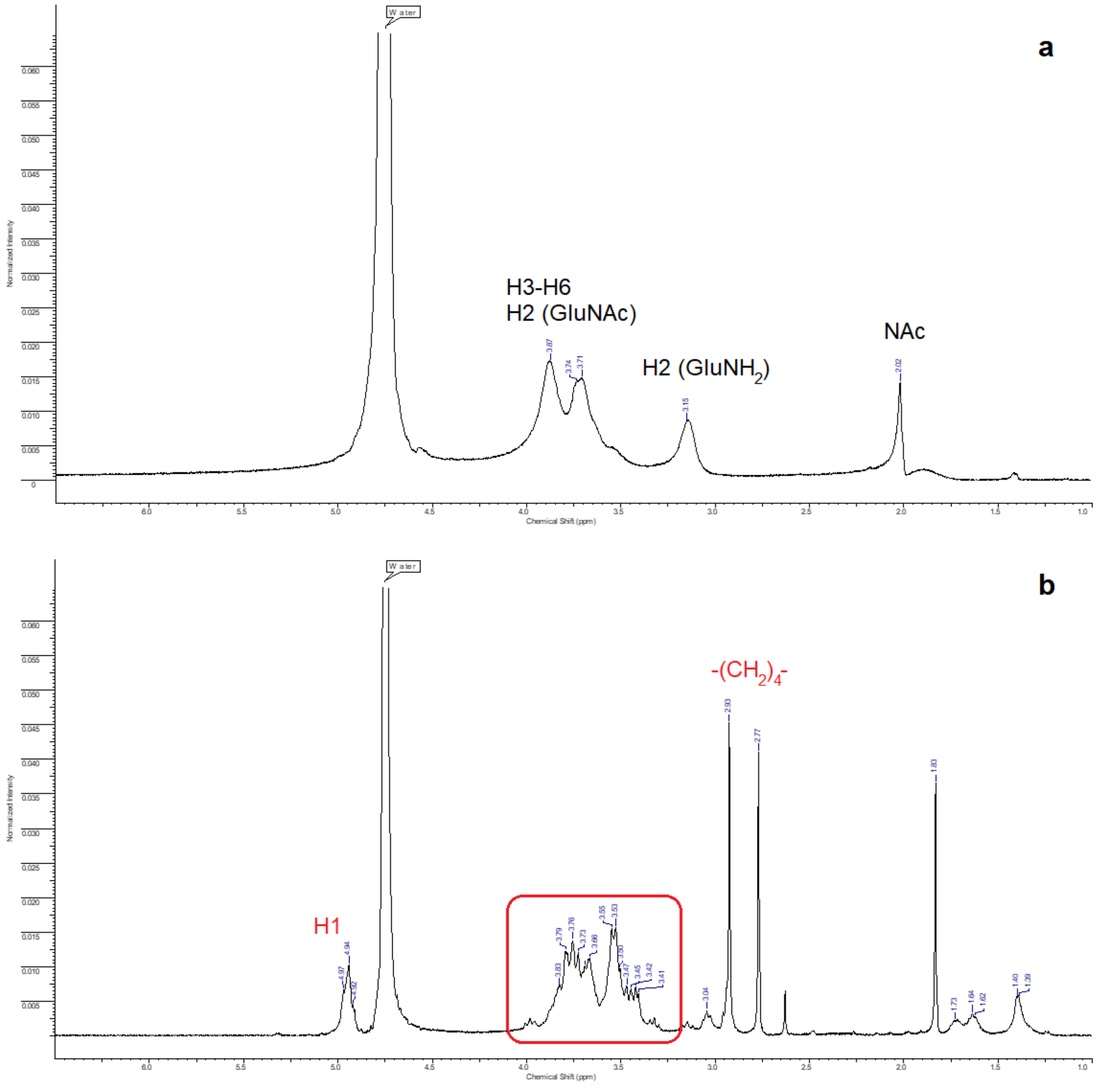

Figure S1. ^{1}H NMR spectra of Chit (**a**) and NH_2-CD-Chit (**b**), D_2O, 400 MHz.
